# Trends in use of neoadjuvant systemic therapy in patients with clinically node-positive breast cancer in Europe: prospective TAXIS study (OPBC-03, SAKK 23/16, IBCSG 57-18, ABCSG-53, GBG 101)

**DOI:** 10.1007/s10549-023-06999-9

**Published:** 2023-06-25

**Authors:** Christoph Tausch, Kavitha Däster, Stefanie Hayoz, Zoltan Matrai, Florian Fitzal, Guido Henke, Daniel R. Zwahlen, Günther Gruber, Frank Zimmermann, Mariacarla Andreozzi, Maite Goldschmidt, Alexandra Schulz, Nadia Maggi, Ramon Saccilotto, Martin Heidinger, Andreas Mueller, Ekaterini Christina Tampaki, Vesna Bjelic-Radisic, Ákos Sávolt, Viktor Smanykó, Daniela Hagen, Dieter J. Müller, Michael Gnant, Sibylle Loibl, Pagona Markellou, Inga Bekes, Daniel Egle, Thomas Ruhstaller, Simone Muenst, Sherko Kuemmel, Conny Vrieling, Rok Satler, Charles Becciolini, Susanne Bucher, Christian Kurzeder, Colin Simonson, Peter M. Fehr, Natalie Gabriel, Robert Maráz, Dimitri Sarlos, Konstantin J. Dedes, Cornelia Leo, Gilles Berclaz, Hisham Fansa, Christopher Hager, Klaus Reisenberger, Christian F. Singer, Giacomo Montagna, Roland Reitsamer, Jelena Winkler, Giang Thanh Lam, Mathias K. Fehr, Tatiana Naydina, Magdalena Kohlik, Karine Clerc, Valerijus Ostapenko, Loïc Lelièvre, Jörg Heil, Michael Knauer, Walter Paul Weber

**Affiliations:** 1 Breast Center Zurich, Zurich, Switzerland; 2 Faculty of Medicine, University of Basel, Basel, Switzerland; 3 SAKK Competence Center, Bern, Switzerland; 4 Hamad Medical Corporation, Department of Oncoplastic Breast Surgery, Doha, Qatar; 5 International Breast Cancer Study Group – a division of ETOP IBCSG Partners Foundation, Bern, Switzerland; 6 Department of Surgery and Comprehensive Cancer Center, Medical University Vienna, Vienna, Austria; 7 ABCSG, Austrian Breast and Colorectal Cancer Study Group, Vienna, Austria; 8 Department of Radiation Oncology, St. Gallen Cantonal Hospital, St. Gallen, Switzerland; 9 Department of Radiation Oncology, Kantonsspital Münsterlingen/Spital Thurgau AG, Münsterlingen, Switzerland; 10 Department of Radiation Oncology, Cantonal Hospital Winterthur, Winterthur, Switzerland; 11 Institute of Radiotherapy, Klinik Hirslanden, Zurich, Switzerland; 12 University of Basel, Basel, Switzerland; 13 Clinic of Radiation Oncology, University Hospital Basel, Basel, Switzerland; 14 Breast Center, University Hospital Basel, Basel, Switzerland; 15 Department of Clinical Research, University Hospital Basel, Basel, Switzerland; 16 Breast Center, Cantonal Hospital Winterthur, Winterthur, Switzerland; 17 Department of Plastic, Reconstructive Surgery and Burn Unit, KAT Athens Hospital and Trauma Center, Athens, Greece; 18 Breast Unit, Helios University Clinic, University Witten, Herdecke, Germany; 19 National Institute of Oncology, Budapest, Hungary; 20 Breast Center, Bethesda Spital AG, Basel, Switzerland; 21 Comprehensive Cancer Center, Medical University of Vienna, Vienna, Austria; 22 German Breast Group, GBG Forschungs GmbH, Neu-Isenburg, Germany; 23 Breast Center, St. Gallen Cantonal Hospital, St. Gallen, Switzerland; 24 Breast Cancer Center Tirol, Department of Gynecology, Medical University Innsbruck, Innsbruck, Austria; 25 Tumor and Breast Center Eastern Switzerland, St. Gallen, Switzerland; 26 Institute of Medical Genetics and Pathology, University Hospital Basel, Basel, Switzerland; 27 Breast Unit, Kliniken Essen-Mitte, Charité, Essen, Germany; 28 Department of Gynecology with Breast Center, Universitätsmedizin Berlin, Berlin, Germany; 29 Department of Radiation Oncology, Hirslanden Clinique des Grangettes, Geneva, Switzerland; 30 Breast Center, Réseau Hospitalier Neuchâtelois, La Chaux-de-Fonds, Switzerland; 31 Breast Center, Cantonal Hospital Lucerne, Lucerne, Switzerland; 32 Department of Gynecology, Centre Hospitalier du Valais Romand (CHVR), Hôpital de Sion, Sion, Switzerland; 33 Breast Center Graubünden, Cantonal Hospital Graubünden, Chur, Switzerland; 34 Breast Center, City Hospital Triemli, Zurich, Switzerland; 35 Department of Oncology, Bacs-Kiskun Country Hospital, Kecskemet, Hungary; 36 Breast Center, Cantonal Hospital Aarau, Aarau, Switzerland; 37 Breast Cancer Center, Zurich Lake, Zurich, Switzerland; 38 Breast Center, Cantonal Hospital Baden, Baden, Switzerland; 39 Breast Center Bern, Lindenhof Group, Bern, Switzerland; 40 Breast Center Zürich, Bethanien & Spital Zollikerberg, Zurich, Switzerland; 41 Department of Gynecology and Obstetrics, City Hospital, Dornbirn, Austria; 42 Department of Gynecology and Obstetrics, Klinikum Wels-Grieskirchen, Wels, Austria; 43 Department of Gynecology and Obstetrics and Comprehensive Cancer Center, Medical University of Vienna, Vienna, Austria; 44 Breast Service, Department of Surgery, Memorial Sloan Kettering Cancer Center, New York, NY USA; 45 Breast Center, Paracelsus Medical University of Salzburg, Salzburg, Austria; 46 Breast Center, University Hospital of Geneva, Geneva, Switzerland; 47 Breast Center Thurgau, Frauenfeld, Switzerland; 48 Spital Limmattal, Schlieren, Switzerland; 49 Breast Center GSMN, Clinique de Genolier, Genolier, Switzerland; 50 Brustzentrum Freiburg, Centre du Sein Fribourg, Fribourg, Switzerland; 51 National Cancer Institute, Vilnius, Lithuania; 52 Breast Center, CHUV, Lausanne, Switzerland; 53 Breast Center Heidelberg, Heidelberg, Germany

**Keywords:** Neoadjuvant systemic therapy, Neoadjuvant chemotherapy, Breast cancer surgery, Clinically node-positive, Tailored axillary surgery, TAXIS trial

## Abstract

**Purpose:**

The aim of this study was to evaluate clinical practice heterogeneity in use of neoadjuvant systemic therapy (NST) for patients with clinically node-positive breast cancer in Europe.

**Methods:**

The study was preplanned in the international multicenter phase-III OPBC-03/TAXIS trial (ClinicalTrials.gov Identifier: NCT03513614) to include the first 500 randomized patients with confirmed nodal disease at the time of surgery. The TAXIS study’s pragmatic design allowed both the neoadjuvant and adjuvant setting according to the preferences of the local investigators who were encouraged to register eligible patients consecutively.

**Results:**

A total of 500 patients were included at 44 breast centers in six European countries from August 2018 to June 2022, 165 (33%) of whom underwent NST. Median age was 57 years (interquartile range [IQR], 48–69). Most patients were postmenopausal (68.4%) with grade 2 and 3 hormonal receptor-positive and human epidermal growth factor receptor 2-negative breast cancer with a median tumor size of 28 mm (IQR 20–40). The use of NST varied significantly across the countries (p < 0.001). Austria (55.2%) and Switzerland (35.8%) had the highest percentage of patients undergoing NST and Hungary (18.2%) the lowest. The administration of NST increased significantly over the years (OR 1.42; p < 0.001) and more than doubled from 20 to 46.7% between 2018 and 2022.

**Conclusion:**

Substantial heterogeneity in the use of NST with HR+/HER2-breast cancer exists in Europe. While stringent guidelines are available for its use in triple-negative and HER2+ breast cancer, there is a need for the development of and adherence to well-defined recommendations for HR+/HER2-breast cancer.

**Supplementary Information:**

The online version contains supplementary material available at 10.1007/s10549-023-06999-9.

## Introduction

Over the last decade, neoadjuvant systemic therapy (NST) has gained considerable therapeutic importance and has been extended to include patients with operable node-positive breast cancer. Although current evidence shows no improvement in survival for these patients, the benefits of locoregional downstaging and response-driven adjuvant therapy continues to drive this shift [1]. The use of NST is not only associated with increased frequency of breast-conserving therapy but also de-escalation of axillary surgery in patients with limited nodal disease [2]. Neoadjuvant therapy, particularly chemotherapy in patients with more aggressive breast cancer subtypes, often converts clinically node-positive (cN+) disease to pathologically node negative (ypN0) [3]. This can therefore be effectively managed with limited lymph node removal with sentinel lymph node (SLN) surgery alone or in combination with imaging-guided localization of the biopsy-proven node (targeted axillary dissection) resulting in lower rates of lymphoedema and other complications [4]. Uncertainties and controversies remain regarding the ideal dose, intensity, duration of proposed NST and treatment options, which potentially leads to a significant amount of heterogeneity in clinical practice between different countries and institutions. For the treating physicians, evidence-based standardization of these practices is critically important. A better understanding of these factors could influence not just the outcome, but also the cost and convenience of the regime. This is essential in the era of quality and value-based medical decision making.

The international multicenter phase-III OPBC-03/TAXIS trial (ClinicalTrials.gov Identifier: NCT03513614) was designed to assess the optimal locoregional management of the axilla in patients with cN+ breast cancer, including patients with residual nodal disease following NST [5]. Its main objective is to show that the combination of tailored axillary surgery (TAS) and axillary radiotherapy (ART) is non-inferior to the current standard of axillary lymph node dissection (ALND) in terms of disease-free survival in the era of effective systemic therapy and extended regional nodal irradiation. The TAXIS study protocol is unique inasmuch as its pragmatic design allows inclusion of patients both in the neoadjuvant and adjuvant setting according to the preferences of the treating physicians and institutions. Therefore, it provides an excellent opportunity to study patterns and trends in the use of NST in different institutions across Europe. The aim of this study was to evaluate the use of NST in patients with clinically node-positive breast cancer in Europe to assess the need for international standardization of NST.

## Methods

This prospective observational cohort study was preplanned within the pragmatic randomized controlled international multicenter phase-III TAXIS trial (OPBC-03/SAKK 23/16/IBCSG 57-18/ABCSG-53/GBG 101; ClinicalTrials.gov Identifier: NCT03513614) to assess trends in use of NST [5]. Patients with cN+ breast cancer were included, defined as nodal disease detected by palpation or imaging at the time of initial diagnosis and histologic or cytologic confirmation of both the primary tumor and lymph node metastasis. According to the pragmatic design, patients can be included in the upfront surgery as well as in the neoadjuvant setting, with mandatory confirmation of residual nodal disease at the time of surgery in the latter setting. Patients with American Joint Commission on Cancer (AJCC) stage IV, cN3c or cN2b breast cancer, contralateral or other tumor malignancy within 3 years, prior axillary surgery except SLN biopsy, or prior axillary radiotherapy were excluded. The patient population in the present study was a priori defined to include the first 500 consecutive randomized patients who were included from August 2018 to June 2022.

The trial was approved by the local ethics committees and was performed in accordance with the requirements of the national regulatory authorities. Written informed consent was obtained from all patients.

### Systemic therapy

In line with the pragmatic TAXIS trial protocol, type of systemic therapy was left to the discretion and preference of the treating physicians and institutions. All drugs used for adjuvant systemic anticancer treatment (if indicated) were locally chosen according to international and/or local guidelines including the sequence of systemic therapy in relation to surgery (neoadjuvant versus adjuvant setting). All drugs used for adjuvant systemic anticancer treatment were systematically recorded. Planning of study visits followed local practice in frequency, interval, and duration. Adjuvant patients were defined as patients who did not receive NST. Investigators were encouraged to enroll all eligible patients consecutively without selecting patients and tumors according to the likelihood of not achieving complete pathological response (pCR) to maintain TAXIS study eligibility.

### Endpoints

Primary endpoint for this study was the rate of patients undergoing NST (proportion of entire TAXIS patient population that underwent NST) [5]. Secondary endpoints included the rate of patients undergoing NST by country, by study site, by stage, and by intrinsic subtype defined by the expression of hormonal receptors (HR) and human epidermal growth factor receptor 2 (HER2).

### Statistical analysis

This project reflects an interim analysis of the TAXIS trial that was pre-planned after 500 patients were randomized (one third of the total sample size). It was planned to gain relevant insight on the use of adjuvant and post-neoadjuvant systemic treatment, which, in turn, may have an impact on the primary endpoint of the main trial (disease-free survival).

Continuous endpoints were summarized using median and interquartile range (IQR) and compared between treatment arms using Wilcoxon rank-sum tests. Categorical endpoints were summarized using frequency counts and percentages and compared between treatment arms using Fisher’s exact tests. Logistic regression was applied to investigate the influence of year of administration of neoadjuvant treatment. Two-tailed tests with a significance level of 0.05 were used. No adjustment was made for multiple testing and all analyses are considered exploratory. All analyses were performed using R version 4.2.1.

## Results

Patient demographics and tumor characteristics in adjuvant versus neoadjuvant setting are shown in Table 1. A total of 750 patients undergoing NST were screened and consented to the TAXIS study, baseline characteristics shown in Supplementary Appendix 1. However, 250 patients were screening failures due to exclusion criteria at the time of surgery, including pathologic complete response (pCR) in 182 patients, no SLN identified in 10, no radiologically identified clip in 38, and other reasons in 20 patients. The baseline characteristics of the 182 patients who were excluded due to nodal pCR are shown in Supplementary Appendix 2. The remaining 500 patients were randomized for the TAXIS trial and included in the present sub-study. Median age was 57 years (interquartile range [IQR]: 48–69 years). Most patients were postmenopausal (68.4%) with grade 2 and 3 HR+HER2-breast cancer with a median tumor size of 28 mm (IQR 20–40). Patients were recruited from 44 breast centers in six countries in Europe. The largest volume of patients was recruited from Switzerland (*n* = 335; 67%), followed by Hungary (*n* = 99; 19.8%).

**Table 1:** Overall patient and tumor characteristics by adjuvant vs neoadjuvant treatment

Characteristic	N = 500^a^	Adjuvant setting, N = 335^b^	Neoadjuvant chemotherapy, N = 151^2^	p-value^c^
Age at registration (years)	57 (48, 69)	61 (50, 72)	50 (43, 58)	< 0.001
Sex				0.073
Female	487 (97.4%)	323 (96.4%)	150 (99.3%)	
Male	13 (2.6%)	12 (3.6%)	1 (0.7%)	
Country				0.001
Austria	29 (5.8%)	13 (3.9%)	11 (7.3%)	
Germany	31 (6.2%)	23 (6.9%)	7 (4.6%)	
Hungary	99 (19.8%)	81 (24.2%)	17 (11.3%)	
Italy	2 (0.4%)	0 (0.0%)	2 (1.3%)	
Lithuania	4 (0.8%)	3 (0.9%)	1 (0.7%)	
Switzerland	335 (67.0%)	215 (64.2%)	113 (74.8%)	
Menopausal status				0.004
Postmenopausal	342 (68.4%)	245 (73.1%)	90 (59.6%)	
Premenopausal	157 (31.4%)	90 (26.9%)	61 (40.4%)	
Unknown	1 (0.2%)			
Tumor type				< 0.001
Invasive ductal	389 (77.8%)	247 (73.7%)	133 (88.1%)	
Invasive lobular	60 (12.0%)	50 (14.9%)	7 (4.6%)	
Other	50 (10.0%)	38 (11.3%)	11 (7.3%)	
Unknown	1 (0.2%)	0 (0.0%)	0 (0.0%)	
Tumor grade				0.063
G1	32 (6.4%)	23 (6.9%)	6 (4.0%)	
G2	294 (58.8%)	206 (61.5%)	80 (53.0%)	
G3	169 (33.8%)	104 (31.0%)	63 (41.7%)	
Unknown	5 (1.0%)	2 (0.6%)	2 (1.3%)	
Type of node positivity				0.7
Node-positivity detected by imaging and non-palpable (iN+)	242 (48.4%)	163 (48.7%)	70 (46.4%)	
Node-positivity palpable (cN1-3)	258 (51.6%)	172 (51.3%)	81 (53.6%)	
Tumor receptor subtype				< 0.001
HR−/HER2−	35 (7.0%)	9 (2.7%)	26 (17.2%)	
HR−/HER2+	5 (1.0%)	2 (0.6%)	2 (1.3%)	
HR+/HER2−	397 (79.4%)	296 (88.4%)	89 (58.9%)	
HR+/HER2+	52 (10.4%)	20 (6.0%)	32 (21.2%)	
Unknown	11 (2.2%)	8 (2.4%)	2 (1.3%)	
Tumor size (mm)	28 (20, 40)	28 (20, 40)	30 (23, 43)	0.028
Unknown	17	12	4	
Type of breast surgery (categorized)				0.7
Breast conserving surgery	293 (58.6%)	193 (57.6%)	90 (59.6%)	
Mastectomy	207 (41.4%)	142 (42.4%)	61 (40.4%)	
Number of lymph nodes removed by TAS	5 (3, 8)	5 (3, 8)	4 (2, 6)	< 0.001
Unknown	7	5	2	
Number of additional lymph nodes removed by ALND after TAS	12 (9, 17)	13 (9, 18)	12 (8, 15)	0.063
Unknown	7	3	4	

*HR* Hormonal receptors, *HER2* Human epidermal growth factor receptor 2, *TAS* Tailored axillary surgery, *ALND* Axillary lymph node dissection

^a^Median (Interquartile range (IQR)); n (%)

^b^14 patients did not receive chemotherapy and were treated with only other systemic therapy modalities i.e., endocrine therapy and immunotherapy

^c^Wilcoxon rank sum test; Fisher’s exact test

Of these 500 patients, 165 patients (33%) were treated with NST, and 335 patients (67%) underwent upfront surgery. Following surgery, a total of 243 patients (48.6%) underwent adjuvant chemotherapy. Patients who underwent neoadjuvant chemotherapy (NACT) were significantly younger and more likely to be premenopausal and having a triple negative or HER2 positive breast cancer than patients who underwent upfront surgery (Table 1).

Among the 165 patients treated with NST, a total of 151 had NACT, 24 received neoadjuvant endocrine therapy (NAET) and 42 immunotherapy (e.g., anti-HER2 therapy; Table 2). While 100 patients were treated with NACT alone, 13 received only NAET and one patient was treated with neoadjuvant double HER2-blockade without chemo- or endocrine therapy.

**Table 2:** Type of neoadjuvant treatment

Characteristic	N = 165^a^
Chemotherapy	151 (91.5%)
Endocrine therapy	24 (14.5%)
Immunotherapy (including anti-HER2)	42 (25.4%)

^a^n (%)

*HER2* Human epidermal growth factor receptor 2

Of 335 patients who did not receive NST, 193 (57.6%) received adjuvant chemotherapy, 102 (30.4%) endocrine therapy alone and 40 patients (12%) received no adjuvant systemic treatment. Of the patients who received adjuvant chemotherapy, 126 (53%) were also treated with other adjuvant treatment modalities.

The use of NACT was significantly influenced by receptor status (Table 3). Among patients with triple negative breast cancers (TNBC) and HER2+ cancers, a significantly larger proportion underwent NACT compared to the HR+/HER2-patients, and significantly more patients with AJCC stage 3 disease received NACT compared to patients with stage 2 disease (33.5% vs. 18.9%; p = 0.007).

**Table 3:** The use of neoadjuvant systemic therapy by intrinsic subtype

Characteristic	HR−/HER2−, N = 35^a^	HR−/HER2+, N = 5^a^	HR+/HER2−, N = 397^a^	HR+/HER2+, N = 52^a^	Unknown, N = 11^a^	p-value^b^
Neoadjuvant treatment administered	26 (74.3%)	3 (60.0%)	101 (25.4%)	32 (61.5%)	3 (27.3%)	< 0.001
Neoadjuvant chemotherapy administered	26 (74.3%)	2 (40.0%)	89 (22.4%)	32 (61.5%)	2 (18.2%)	< 0.001

^a^n (%)

^b^Fisher’s exact test

*HR* Hormonal receptors, *HER2* Human epidermal growth factor receptor 2

Use of NST varied significantly across countries (Table 4) (p < 0.001). Austria had the highest overall number of patients undergoing NST and Hungary the lowest. The use of NACT across countries did not differ significantly in stage 2 (p = 0.2) disease, but differences were significant for stage 3 disease (p = 0.008). Finally, the administration of NST increased significantly over the years (OR 1.42 95% CI 1.18–1.71; p < 0.001). The proportion of patients receiving NST more than doubled from 20 to 46.7% from 2018 to 2022 (Table 5, Supplementary Appendix 3).

**Table 4:** The use of neoadjuvant systemic therapy by country

Characteristic	Austria, N = 29^a^	Germany, N = 31^a^	Hungary, N = 99^a^	Italy, N = 2^a^	Lithuania, N = 4^a^	Switzerland, N = 335^a^	p-value^b^
Neoadjuvant treatment administered	16 (55.2%)	8 (25.8%)	18 (18.2%)	2 (100.0%)	1 (25.0%)	120 (35.8%)	< 0.001
Chemotherapy	11 (37.9%)	7 (22.6%)	17 (17.2%)	2 (100.0%)	1 (25.0%)	113 (33.7%)	0.004
Endocrine therapy	5 (17.2%)	1 (3.2%)	1 (1.0%)	0 (0.0%)	0 (0.0%)	17 (5.1%)	0.033
Immunotherapy	5 (17.2%)	2 (6.5%)	3 (3.0%)	2 (100.0%)	0 (0.0%)	30 (9.0%)	0.003
No neoadjuvant therapy	13 (44.8%)	23 (74.2%)	81 (81.8%)	0 (0.0%)	3 (75.0%)	215 (64.2%)	< 0.001

^a^n (%)

^b^Fisher’s exact test

**Table 5:** The administration of neoadjuvant treatment by year

Characteristic	2018, N = 20^a^	2019, N = 144^a^	2020, N = 222^a^	2021, N = 54^a^	2022, N = 60^a^	p-value^b^
Neoadjuvant treatment administered	4 (20.0%)	36 (25.0%)	72 (32.4%)	25 (46.3%)	28 (46.7%)	0.004
Neoadjuvant chemotherapy administered	3 (15.0%)	33 (22.9%)	65 (29.3%)	23 (42.6%)	27 (45.0%)	0.003

^a^n (%)

^b^Pearson’s Chi-squared test

## Discussion

Careful patient selection for NST is a key component of best practice in breast cancer management. The present study shows that while there was a significant increase in the use of NST, there was also substantial heterogeneity by country and by study site, primarily in patients with HR+/HER2-breast cancer. Several findings were expected. For example, premenopausal patients were more likely to be treated with NACT in comparison to postmenopausal patients. It has been well established that chemotherapy in premenopausal women under the age of 50 can improve disease-free survival [6, 7]. Recent exploratory analyses using multigene assays have suggested that the use of chemotherapy was associated with some benefit for women 50 years of age or younger with node-negative disease and midrange Oncotype DX 21-gene recurrence score of 16 to 25, as well as most patients with 1–3 positive nodes and a recurrence score ≤ 25 [8]. Despite these studies only including patients where chemotherapy was administered in an adjuvant setting, this data is often extrapolated to favor the use of NST worldwide. The use of multigene assays in the neoadjuvant setting is slowly gaining popularity. Oncotype DX is the most extensively studied assay in this context, with higher rates of pCR in patients with a high recurrence score compared to patients with low to intermediate scores [9]. Furthermore, in node-positive patients, higher recurrence score results were significantly associated with the likelihood of pCR in the axilla [10]. However, we did not collect data on the use of genomic tests to refine indications for neoadjuvant therapy in this study and, hence, were not capable of evaluating the proportion of patients undergoing NACT based on genomic high risk.

Interestingly, in our study, only 13 patients were solely treated with NAET (tamoxifen 31% and aromatase inhibitors 61%). The sample size is too small to perform a formal comparison between baseline and tumor characteristics of patients who underwent NAET alone and patients who underwent neoadjuvant chemotherapy. However, we added a table as appendix 4 showing the baseline characteristics by use of NAET or neoadjuvant chemotherapy, indicating the expected selection of patients with small G1-2 hormonal receptor positive tumors. Unfortunately, data collection on treatment duration was incomplete at the date of data cut-off and was thus not reported. The use of NAET may gain popularity in the future, as the use of multigene assays in the neoadjuvant setting have been shown to successfully predict cancer response to NAET [11]. The largest of these trials, the TransNEOS trial prospectively validated the role of recurrence score testing in predicting clinical response after 6 months of neoadjuvant letrozole in patients with ER+/HER2-breast cancer. The authors showed that low Oncotype DX recurrence scores are associated with higher clinical response rates in these patients [12].

Among patients treated with NST in our study, the majority had HR+/HER2-breast cancer (61%). This does not seem to fully reflect European NST practice given that some investigators may have selected these patients for pre-registration in the study because patients with triple negative and HER2+ breast cancer have a higher likelihood of pCR, which excludes them from the TAXIS trial. This will be discussed more in detail in the limitation section below. When comparing patients who underwent upfront surgery to patients who had NST, the ones with triple negative and HER2+ breast cancer were more likely to receive NST compared to primary surgery. This is expected given that HER2-positivity mandates the use of targeted HER2 therapy, which is usually combined with chemotherapy [13], and associated with an increased pCR rate [14, 15]. This is an important prognostic marker as a pCR following NST has been shown to translate into a sustained benefit in event-free survival [14]. Furthermore, response-driven chemotherapy became standard care in women with residual triple negative and HER2+ disease following NST after publication of the landmark trials showing a relevant benefit for the administration of adjuvant capecitabine and trastuzumab emtansine, respectively [16, 17]. Therefore, patients with these subtypes received standard NST except for the beginning of the study.

Austria had a high proportion of patients undergoing NST recruited to the study (55%). Participating sites are members of the Austrian Breast & Colorectal Cancer Study Group (ABCSG). The ABCSG has facilitated standardization of diagnostics and therapy in breast cancer throughout Austria providing patients with the latest and best possible treatments including participation in clinical trials, which may have accentuated the use of NST in Austria. Finally, there was a difference observed in the increased use of NACT across countries in AJCC stage 3 disease, but not in stage 2 disease. In the present study, patients with stage 2 disease were more likely to have smaller breast cancers and therefore the use of NACT was less likely to improve the ability to conserve the breast by local down-staging, which may have contributed to that finding.

NST primarily consisted of NACT and was increasingly used over the study period; in fact, its use more than doubled from 2018 to 2022*.* According to the pragmatic trial design, indications for systemic therapy including timing (neoadjuvant versus adjuvant) were left at the discretion of the local investigators. This was necessary to ensure applicability of the generated data to the participating institutions, while in explanatory trials, uniformity of treatment regimens is achieved by standardization in the study protocol. Despite this pragmatic approach, a significant increase in the use of NACT was observed in every country except for Lithuania and Italy with the lowest numbers of patients. This reflects the change in practice in many centers where NACT became increasingly well received by patients and clinicians alike over the last few years. As we entered the era for de-escalation of surgical treatment in patients with breast cancer, NACT is increasingly used to reduce the tumor size allowing for breast conservation and better aesthetic results and for downstaging the axilla in node positive patients [2]. Following NACT, up to 60% of patients with HER2+ and 48% with TNBC breast cancer with initially node-positive disease showed a pCR in the axilla [3]. With the implementation of the SLN procedure and targeted axillary dissection to determine nodal pCR, and low axillary recurrence rates without ALND, and its endorsement by clinical guidelines, NACT gained further popularity over the last few years since these patients who converted to clinically node-negative following NACT can be spared ALND [18–25].

### Limitations

**Fig. 1:**
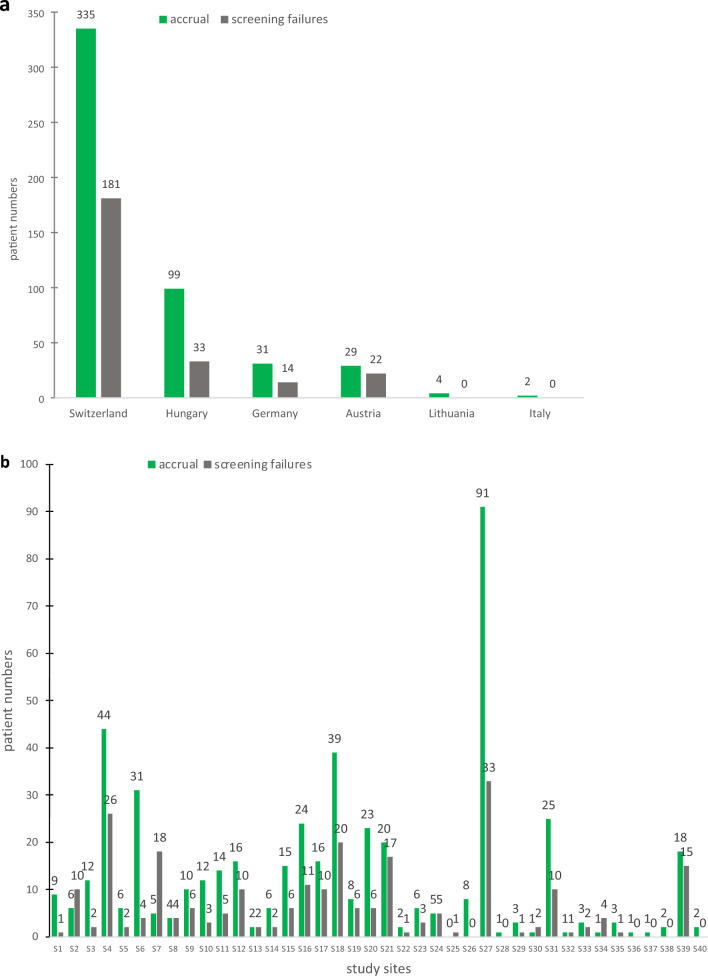
**a** Accrual and screening failures per country. **b** Accrual and screening failures

Our study excluded a large number of patients without residual axillary disease following NST as pCR screening failures. Importantly, investigators were encouraged to pre-register every eligible patient consecutively irrespective of intrinsic breast cancer subtype. However, we found a differing percentage of patients with screening failures by country (Fig. 1a) and by study site (Fig. 1b). In addition, the over-representation of patients with HR+/HER2-disease (79.4%, Table 1) further suggests selection bias toward pre-registration of patients with a lower likelihood of pCR, which, in turn, reduced the number of screening failures that were not reimbursed by the patient fee. As in most pragmatic trials, the risk spectrum of included patients is significant since the majority of patients who undergo the procedure under investigation- in this case axillary dissection-should be included. Skepticism among clinicians may hamper acceptability of such protocols at both ends of the risk spectrum. For example, patients with low volume disease burden may be selectively omitted because surgeons may consider axillary dissection as overtreatment in patients with only one or two positive nodes that otherwise fulfil the Z0011 criteria. This is particularly applicable to patients without palpable disease when nodal metastases are detected solely by ultrasound. On the other hand, surgeons may be reluctant to omit axillary dissection in patients with residual palpable disease after neoadjuvant chemotherapy who are randomized into the axillary radiation arm. To account for that potential selection bias, method of detection of nodal disease (palpable versus imaging only) is used as stratification factor in the TAXIS trial. Furthermore, the lower number of patients included outside of Switzerland limits the generalizability of these findings. Moreover, the analysis might be biased as not all breast centers in these countries included patients into the trial.

## Conclusion

NST in patients with luminal breast cancer is adequately represented in the TAXIS population. However, substantial heterogeneity in the use of NST in patients with HR+/HER2-breast cancer exists in Europe. While stringent guidelines are available for the use of NST in triple-negative and HER2+ breast cancer, the presented data show the need for the development of and adherence to such well-defined recommendations also for patients with HR+/HER2-breast cancer.

## Supplementary Information

Below is the link to the electronic supplementary material.

Supplementary file1 (DOCX 25 kb)

## Data Availability

Prof. Weber had full access to all the data generated during the current study and takes responsibility for the integrity of the data and the accuracy of the data analysis.

## References

[1] Mauri D, Pavlidis N, Ioannidis JPA (2005). Neoadjuvant versus adjuvant systemic treatment in breast cancer: a meta-analysis. J Natl Cancer Inst.

[2] Asselain B, Barlow W, Bartlett J (2018). Long-term outcomes for neoadjuvant versus adjuvant chemotherapy in early breast cancer: meta-analysis of individual patient data from ten randomised trials. Lancet Oncol.

[3] Samiei S, Simons JM, Engelen SME (2021). Axillary pathologic complete response after neoadjuvant systemic therapy by breast cancer subtype in patients with initially clinically node-positive disease: a systematic review and meta-analysis. JAMA Surg.

[4] El Hage CH, Headon H, El Tokhy O (2016). Is sentinel lymph node biopsy a viable alternative to complete axillary dissection following neoadjuvant chemotherapy in women with node-positive breast cancer at diagnosis? An updated meta-analysis involving 3398 patients. Am J Surg.

[5] Henke G, Knauer M, Ribi K (2018). Tailored axillary surgery with or without axillary lymph node dissection followed by radiotherapy in patients with clinically node-positive breast cancer (TAXIS): study protocol for a multicenter, randomized phase-III trial. Trials.

[6] Cathcart-Rake EJ, Ruddy KJ, Bleyer A, Johnson RH (2021). Breast cancer in adolescent and young adult women under the age of 40 years. JCO Oncol Pract.

[7] Albain K, Anderson S, Arriagada R (2012). Comparisons between different polychemotherapy regimens for early breast cancer: meta-analyses of long-term outcome among 100 000 women in 123 randomised trials. Lancet.

[8] Sparano JA, Gray RJ, Makower DF (2018). Adjuvant chemotherapy guided by a 21-gene expression assay in breast cancer. N Engl J Med.

[9] Boland MR, Al-Maksoud A, Ryan EJ (2021). Value of a 21-gene expression assay on core biopsy to predict neoadjuvant chemotherapy response in breast cancer: systematic review and meta-analysis. Br J Surg.

[10] Pardo JA, Fan B, Mele A (2021). The role of oncotype DX® recurrence score in predicting axillary response after neoadjuvant chemotherapy in breast cancer. Ann Surg Oncol.

[11] Davey MG, Ryan J, Boland MR (2021). Clinical utility of the 21-gene assay in predicting response to neoadjuvant endocrine therapy in breast cancer: a systematic review and meta-analysis. Breast.

[12] Iwata H, Masuda N, Yamamoto Y (2019). Validation of the 21-gene test as a predictor of clinical response to neoadjuvant hormonal therapy for ER+, HER2-negative breast cancer: the TransNEOS study. Breast Cancer Res Treat.

[13] Burstein HJ, Curigliano G, Loibl S (2019). Estimating the benefits of therapy for early-stage breast cancer: the St. GALLEN International Consensus Guidelines for the primary therapy of early breast cancer 2019. Ann Oncol.

[14] Gianni L, Eiermann W, Semiglazov V (2014). Neoadjuvant and adjuvant trastuzumab in patients with HER2-positive locally advanced breast cancer (NOAH): follow-up of a randomised controlled superiority trial with a parallel HER2-negative cohort. Lancet Oncol.

[15] von Minckwitz G, Procter M, de Azambuja E (2017). Adjuvant pertuzumab and trastuzumab in early HER2-positive breast cancer. N Engl J Med.

[16] von Minckwitz G, Huang C-S, Mano MS (2019). Trastuzumab emtansine for residual invasive HER2-positive breast cancer. N Engl J Med.

[17] Masuda N, Lee S-J, Ohtani S (2017). Adjuvant capecitabine for breast cancer after preoperative chemotherapy. N Engl J Med.

[18] Boughey JC, Suman VJ, Mittendorf EA (2013). Sentinel lymph node surgery after neoadjuvant chemotherapy in patients with node-positive breast cancer. JAMA.

[19] Kuehn T, Bauerfeind I, Fehm T (2013). Sentinel-lymph-node biopsy in patients with breast cancer before and after neoadjuvant chemotherapy (SENTINA): a prospective, multicentre cohort study. Lancet Oncol.

[20] Burstein HJ, Curigliano G, Thürlimann B (2021). Customizing local and systemic therapies for women with early breast cancer: the St. Gallen international consensus guidelines for treatment of early breast cancer 2021. Ann Oncol.

[21] Boughey JC, Ballman KV, Le-Petross HT (2016). Identification and resection of clipped node decreases the false-negative rate of sentinel lymph node surgery in patients presenting with node-positive breast cancer (T0–T4, N1–N2) who receive neoadjuvant chemotherapy. Ann Surg.

[22] Boileau JF, Poirier B, Basik M (2015). Sentinel node biopsy after neoadjuvant chemotherapy in biopsy-proven node-positive breast cancer: the SN FNAC study. J Clin Oncol.

[23] Laws A, Hughes ME, Hu J (2019). Impact of residual nodal disease burden on technical outcomes of sentinel lymph node biopsy for node-positive (cN1) breast cancer patients treated with neoadjuvant chemotherapy. Ann Surg Oncol.

[24] Mamtani A, Barrio AV, King TA (2016). How often does neoadjuvant chemotherapy avoid axillary dissection in patients with histologically confirmed nodal metastases? Results of a prospective study. Ann Surg Oncol.

[25] Nguyen TT, Hoskin TL, Day CN (2018). Decreasing use of axillary dissection in node-positive breast cancer patients treated with neoadjuvant chemotherapy. Ann Surg Oncol.

