# Supplementary material for: Trends in use of neoadjuvant systemic therapy in patients with clinically node-positive breast cancer in Europe: prospective TAXIS study (OPBC-03, SAKK 23/16, IBCSG 57-18, ABCSG-53, GBG 101)
Source: Breast Cancer Res Treat. 2023 Jun 25;201(2):215–25. doi: 10.1007/s10549-023-06999-9 (PMC10361860; doi:10.1007/s10549-023-06999-9)
Supplement: Supplementary file 1 — Supplementary file1 (DOCX 25 kb) [file 10549_2023_6999_MOESM1_ESM.docx]

Appendix 1: Patient demographics for the overall 750 patients screened for TAXIS.

| Characteristic | N = 750^1^ |
| --- | --- |
| Age at registration (years) | 56 (46, 68) |
| Sex |  |
| Female | 737 (98.3%) |
| Male | 13 (1.7%) |
| Tumor receptor subtype |  |
| HR-/HER2- | 85 (11.3%) |
| HR-/HER2+ | 42 (5.6%) |
| HR+/HER2- | 468 (62.4%) |
| HR+/HER2+ | 125 (16.7%) |
| Unknown | 30 (4.0%) |
| Country |  |
| Austria | 51 (6.8%) |
| Germany | 45 (6.0%) |
| Hungary | 132 (17.6%) |
| Italy | 2 (0.3%) |
| Lithuania | 4 (0.5%) |
| Switzerland | 516 (68.8%) |
| Tumor size (mm) | 29 (20, 41) |
| Unknown | 31 |
| ^1^Median (IQR); n (%) |  |

Appendix 2: The baseline characteristics of patients excluded from the study due to pCR.

| Characteristic | N = 182^1^ |
| --- | --- |
| Age at registration (years) | 54 (45, 62) |
| Sex |  |
| Female | 182 (100.0%) |
| Male | 0 (0.0%) |
| Tumor receptor subtype |  |
| HR-/HER2- | 42 (23.1%) |
| HR-/HER2+ | 33 (18.1%) |
| HR+/HER2- | 27 (14.8%) |
| HR+/HER2+ | 65 (35.7%) |
| Unknown | 15 (8.2%) |
| Year |  |
| 2018 | 4 (2.2%) |
| 2019 | 57 (31.3%) |
| 2020 | 84 (46.2%) |
| 2021 | 20 (11.0%) |
| 2022 | 17 (9.3%) |
| Country |  |
| Austria | 17 (9.3%) |
| Germany | 11 (6.0%) |
| Hungary | 23 (12.6%) |
| Italy | 0 (0.0%) |
| Lithuania | 0 (0.0%) |
| Switzerland | 131 (72.0%) |
| Tumor size (mm) | 30 (21, 45) |
| Unknown | 8 |
| ^1^Median (IQR); n (%) | |

Appendix 3: The administration of neoadjuvant treatment by year.
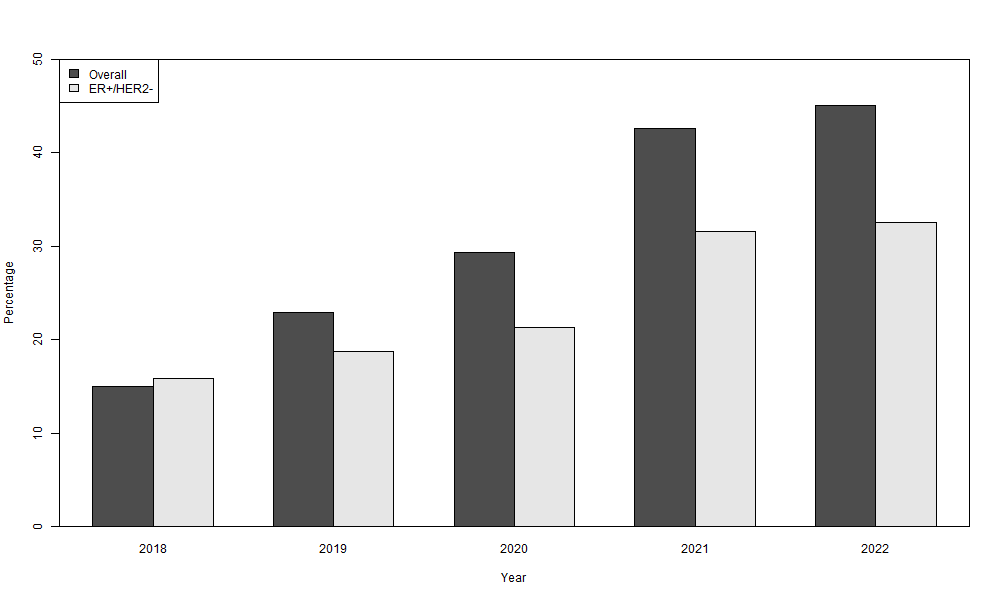


Appendix 4: The baseline characteristics by use of NAET or neoadjuvant chemotherapy.

| Characteristic | Neoadjuvant endocrine therapy only, N = 13^1^ | Neoadjuvant chemotherapy, N = 151^1^ |
| --- | --- | --- |
| Age at registration (years) | 59 (51, 69) | 50 (43, 58) |
| Sex |  |  |
| Female | 13 (100.0%) | 150 (99.3%) |
| Male | 0 (0.0%) | 1 (0.7%) |
| Country |  |  |
| Austria | 5 (38.5%) | 11 (7.3%) |
| Germany | 1 (7.7%) | 7 (4.6%) |
| Hungary | 1 (7.7%) | 17 (11.3%) |
| Italy | 0 (0.0%) | 2 (1.3%) |
| Lithuania | 0 (0.0%) | 1 (0.7%) |
| Switzerland | 6 (46.2%) | 113 (74.8%) |
| Menopausal status |  |  |
| Postmenopausal | 6 (46.2%) | 90 (59.6%) |
| Premenopausal | 6 (46.2%) | 61 (40.4%) |
| Unknown | 1 (7.7%) |  |
| Tumor type |  |  |
| Invasive ductal | 8 (61.5%) | 133 (88.1%) |
| Invasive lobular | 3 (23.1%) | 7 (4.6%) |
| Other | 1 (7.7%) | 11 (7.3%) |
| Unknown | 1 (7.7%) | 0 (0.0%) |
| Tumor grade |  |  |
| G1 | 3 (23.1%) | 6 (4.0%) |
| G2 | 8 (61.5%) | 80 (53.0%) |
| G3 | 1 (7.7%) | 63 (41.7%) |
| Unknown | 1 (7.7%) | 2 (1.3%) |
| Type of node positivity |  |  |
| Node-positivity detected by imaging and non-palpable (iN+) | 9 (69.2%) | 70 (46.4%) |
| Node-positivity palpable (cN1-3) | 4 (30.8%) | 81 (53.6%) |
| Tumor receptor subtype |  |  |
| HR-/HER2- | 0 (0.0%) | 26 (17.2%) |
| HR-/HER2+ | 0 (0.0%) | 2 (1.3%) |
| HR+/HER2- | 12 (92.3%) | 89 (58.9%) |
| HR+/HER2+ | 0 (0.0%) | 32 (21.2%) |
| Unknown | 1 (7.7%) | 2 (1.3%) |
| Tumor size (mm) | 21 (12, 28) | 30 (23, 43) |
| Unknown | 1 | 4 |
| Type of breast surgery (categorized) |  |  |
| Breast conserving surgery | 9 (69.2%) | 90 (59.6%) |
| Mastectomy | 4 (30.8%) | 61 (40.4%) |
| Number of lymph nodes removed by TAS | 7 (5, 7) | 4 (2, 6) |
| Unknown | 0 | 2 |
| Number of additional lymph nodes removed by ALND after TAS | 17 (12, 19) | 12 (8, 15) |
| Unknown | 10 | 4 |

^1^Median (IQR); n (%)
